# The relationship between daytime napping and obesity: a systematic review and meta-analysis

**DOI:** 10.1038/s41598-023-37883-7

**Published:** 2023-07-26

**Authors:** Zixin Cai, Yan Yang, Jingjing Zhang, Yu Liu

**Affiliations:** 1 National Clinical Research Center for Metabolic Diseases, Metabolic Syndrome Research Center, Key Laboratory of Diabetes Immunology, Ministry of Education, and Department of Metabolism and Endocrinology, The Second Xiangya Hospital of Central South University, Changsha, 410011 Hunan China; 2 Department of Nephrology, Hunan Key Laboratory of Kidney Disease and Blood Purification, The Second Xiangya Hospital at Central South University, Changsha, 410011 China

**Keywords:** Endocrinology, Health care

## Abstract

Daytime napping, a habit widely adopted globally, has an unclear association with obesity. In this study, we executed a meta-analysis to explore the relationship between daytime napping and obesity. We conducted a comprehensive search of the PubMed, Embase, Cochrane Library, Scopus, PsycINFO, and Web of Science databases for pertinent articles published up to April 2023. Random-effects models were utilized to calculate odds ratios (ORs) with 95% confidence intervals (CIs), and we assessed the heterogeneity of the included studies using the *I*^2^ statistic. To explore potential sources of heterogeneity, subgroup analyses were performed. The methodological quality of the studies was evaluated using the Newcastle–Ottawa Scale (NOS), and funnel plots were employed to detect any publication bias. Sensitivity analyses were conducted by sequentially omitting each study. We conducted a meta-analysis of twelve studies that included one each from the UK and Spain, five from the USA, and five from China, totalling 170,134 participants, to probe the association between napping and obesity. The pooled analysis suggested a higher risk of obesity in individuals who nap (OR: 1.22 [1.10–1.35], p < 0.001,* I*^2^ = 87%) compared to non-nappers. The meta-analysis results revealed variations in the summary ORs for studies conducted in China, Spain, the USA, and the UK. The ORs for China, Spain, the USA, and the UK were 1.05 (95% CI 0.90–1.23), 9.36 (95% CI 4.74–18.45), 1.27 (95% CI 1.10–1.47), and 1.39 (95% CI 1.32–1.47), respectively. A subgroup analysis based on age within the American population disclosed that napping in both adults and children heightened obesity incidence. A subgroup analysis based on nap duration found a significant rise in obesity occurrence when nap duration exceeded one hour, but no clear relationship emerged when nap duration was less than 1 h. In a subgroup analysis based on the definition of obesity, napping did not demonstrate a significant relationship with obesity when diagnostic criteria set obesity at a BMI of 25 or above. However, when the criteria were set at a BMI of 28 or 30 or more, napping significantly increased obesity risk. Our meta-analysis indicates a positive association between daytime napping and the risk of obesity. However, given the limited number of included studies, potential confounding factors might not have been fully addressed. Future well-designed prospective studies are required to further investigate this relationship. Large-scale studies are necessary to confirm our findings and elucidate the underlying mechanisms that drive these associations and causation.

## Introduction

Did you know that over 1.9 billion adults worldwide are overweight, and 650 million of them are obese^1^? These staggering numbers reflect a global health crisis that has far-reaching consequences. Obesity, characterized by excessive body fat accumulation, has become a pressing issue affecting individuals of all ages and socioeconomic backgrounds. It is not merely a cosmetic concern but a complex condition with significant implications for overall health and well-being. Obesity poses numerous health risks and is associated with a range of chronic diseases, including diabetes^2^, cardiovascular diseases^3^, and certain types of cancer^4^. Obesity significantly contributes to morbidity and reduced life expectancy, with cardiovascular disease (CVD) and cancer accounting for the greatest mortality risk^5^. Moreover, it places an enormous burden on healthcare systems and contributes to rising healthcare costs. The causes of obesity are multifactorial, involving a combination of genetic^6^, environmental^7^, and behavioral factors^8^. Unhealthy dietary patterns, such as high consumption of processed foods and sugary beverages, coupled with sedentary lifestyles, play a pivotal role in the development and progression of obesity^9^. Efforts to address this global epidemic require a comprehensive approach encompassing individual behavior change, community interventions, and policy initiatives. Encouraging healthier eating habits, promoting physical activity, and fostering supportive environments are crucial steps towards curbing the obesity crisis. Additionally, raising awareness about the long-term health consequences of obesity and the importance of preventive measures is essential for promoting a healthier future.

As researchers strive to understand the complex factors contributing to obesity, recent studies have begun to explore the potential influence of daytime napping on weight management. In our modern fast-paced society, sleep deprivation has become increasingly common, with many individuals not getting enough restorative sleep at night. This sleep deficit has led to a rise in daytime napping as a way to compensate for the lack of sleep^10^. While daytime napping has been linked to various health benefits, including improved cognitive function and mood^11^, its association with obesity remains a subject of investigation. Previous research on the relationship between daytime napping and obesity risk has generated conflicting and inconsistent findings. Some studies have indicated a potential association between increased daytime napping and a higher risk of obesity. For instance, a study found that excessive daytime napping was linked to a higher body mass index (BMI) and increased odds of being obese^12^. However, other studies have reported different outcomes, suggesting no significant association or even a possible inverse relationship between daytime napping and obesity risk^13^. In addition, other studies have reported that daytime napping is associated with a lower risk of obesity^14^. These conflicting findings may be attributed to differences in study designs, sample sizes, and measurement methods across various studies. Considering the inconsistency and limitations of previous research, it is necessary to conduct a meta-analysis to better understand the relationship between daytime napping and the risk of obesity. This study aims to address these knowledge gaps through a comprehensive meta-analysis of existing literature, leveraging large and diverse sample sizes, objective measurements, and meticulous control of potential confounding variables. By elucidating this relationship, we can enhance our understanding of the potential impact of daytime napping on obesity risk and provide valuable insights for public health strategies aimed at addressing the global obesity epidemic.

This study aims to examine the potential relationship between daytime napping and obesity risk. By investigating whether there is a correlation between napping duration and body weight, we hope to shed light on the role of napping in weight management and contribute to the growing body of research on sleep and obesity. By understanding the impact of daytime napping on obesity, we can potentially develop more effective strategies for weight management and public health interventions. This research is crucial in addressing the global obesity epidemic and promoting healthier lifestyles.

## Methods

### Search strategy

We conducted a systematic review following the Preferred Reporting Items for Systematic Reviews and Meta-Analyses (PRISMA) guidelines^15^. The PRISMA checklist was presented as Supplemental file 1. A comprehensive search was performed in the PubMed, Embase, Cochrane Library, Scopus, PsycINFO and Web of Science databases from their inception until April 2023 to identify studies examining the association between napping and/or obesity. The following search terms were used: “siesta” OR “napping” OR “nap” OR "afternoon" OR "midday" OR "dozing" OR "daytime sleep" AND "obesity" OR "overweight" OR "BMI". Additionally, we manually searched the references of eligible studies and identified reviews to find other relevant studies.

### Study selection

To be eligible for inclusion, studies had to meet the following criteria: (1) be original research articles, (2) examine the association between daytime napping and prevalent and/or incident obesity as the outcome of interest, (3) report odds ratios (ORs) with corresponding 95% confidence intervals (CIs), (4) include human participants, and (5) be written in English. Non-original research articles were excluded from the meta-analysis. The quality of the included studies was evaluated using the Newcastle Ottawa Scale (NOS) for cross-sectional or cohort studies^16^. Two reviewers (ZC and YY) screened the titles, abstracts, and full-texts of all potentially eligible studies. If a consensus could not be reached, any disagreements between reviewers were resolved by a third reviewer (JZ).

### Data extraction

We collected information using a form that included study details such as author, year of publication, country of origin, and study type, as well as participant characteristics such as the size of the total study population, age, and percentage of male participants. We also collected information on the definitions of daytime napping and obesity.

### Quality assessment

To assess the quality of the included studies, two reviewers (ZC and YY) independently evaluated them using the Newcastle Ottawa Scale (NOS) quality assessment tool. The NOS assesses studies based on three aspects: the selection of exposed and unexposed participants, the comparability of the groups, and the evaluation of the outcomes. A score range of 0 to 9 was used, and we considered studies with an NOS score > 6 to be of high quality. Article quality was judged by the NOS checklist, and total evaluation scores of each study regarding selection, comparability and outcome ascertainment are summarized in Supplemental table 1.

### Statistical analyses

To measure the association between daytime napping and obesity risk, we used the overall odds ratio (OR) score. Heterogeneity was evaluated using the *I*^2^ statistic^17^. Significant heterogeneity was defined as *p* < 0.1 and *I*^2^ > 50%^18^. If significant heterogeneity was found, we used the inverse variance random effects model to combine the ORs. Otherwise, a fixed-effect model was used. We assessed publication bias using a funnel plot and both Begg’s and Egger’s tests. A *p* value of less than 0.05 indicated significant publication bias^19^. We also conducted subgroup analyses based on study type, sample size, and country. The meta-analysis was performed using Stata/SE (StataCorp LP, Release 13, College Station, TX, USA).

## Results

### Literature search

**Figure 1:**
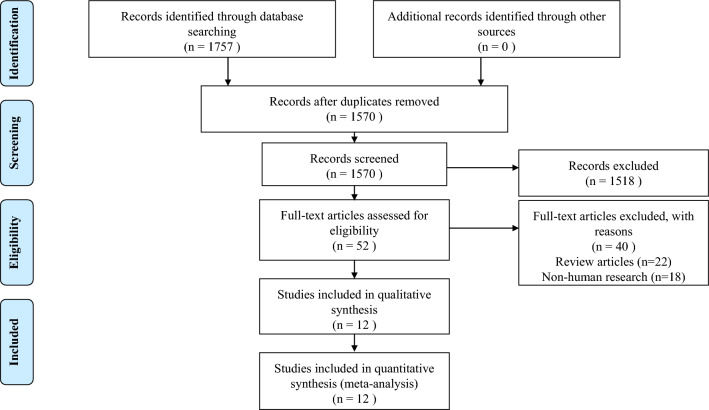
Study election.

Figure 1 summarizes the results of the search and study selection processes, which yielded a total of 1757 articles (590 from PubMed, 228 from Embase, 0 from the Cochrane Library, and 939 from Web of Science). We manually searched the references cited in selected articles and review articles, but did not find any new eligible articles. After excluding repeated citations (n = 187), we screened the titles and abstracts of the remaining 1570 articles, and excluded 22 reviews/comments and 18 articles describing non-human research. Finally, we retrieved the full texts of 52 articles and excluded 40 of them for not meeting the inclusion criteria. Ultimately, the remaining twelve studies met the inclusion criteria and were included in the meta-analysis.

### Study characteristics

Table 1 presents the characteristics of 12 studies that were conducted on a total of 170,134 participants. Out of these, five studies were conducted in China^14,20–23^, five were conducted in the USA^12,13,24–26^, while the remaining two were conducted in the United Kingdom (UK)^27^and Spain^28^. All the studies included both men and women, and nine of them were published within the last 5 years, while the other three were published before 2016. The definitions of napping and obesity used in each study are provided in Table 1. Most studies used questionnaires to collect data on the frequency of napping. The studies included seven cross-sectional studies and five cohort studies that analyzed the relationship between daytime napping and the risk of obesity. All the studies included in this analysis had a quality score of over 6, indicating good quality, as assessed by the Newcastle–Ottawa Scale (NOS). Overall, the studies in Table 1 provide a good representation of the association between daytime napping and obesity risk across different countries and study designs.

**Table 1:** Characteristics of available studies on the relationship between daytime napping and obesity.

Number	Author	Year	Country	Sample size	Studies type	Age	Male participants	Definitions of daytime napping	Time of napping	Definitions of obesity	NOS
1	Diaozhu Lin	2014	China	8547	Cross-sectional study	56.0 ± 8.0	28.20%	Self-reported	0.1-1 h; > 1 h	BMI ≥ 28	8
2	Kui Peng	2017	China	8559	Cross-sectional study	58.5 ± 9.0	36.30%	Self-reported	≤ 0.5 h; > 0.5 h	BMI ≥ 28	7
3	José S. Loredo	2019	Spain	2156	Cross-sectional study	18–64	NA	Self-reported	> 15 min	BMI ≥ 30	7
4	Yue Leng	2017	USA	2675	Cohort study	84.5 ± 3.7	NA	Self-reported	> 1 h	NA	8
5	Janice F. Bell	2010	USA	1930	Cohort study	0–13	50.00%	Parental report	< 0.5 h	NA	7
6	Xueyin Zhao	2020	China	3236	Cohort study	52.5 ± 13.2	37.50%	Self-report	≤ 0.5 h;0.5–1 h; > 1 h	BMI ≥ 25	8
7	Lara Nasreddine	2018	USA	1047	Cross-sectional study	15.9 ± 1.9	62.60%	Self-report	NA	MUO or MHO	6
8	Carlos Celis-Morales	2017	UK	119,859	Cross-sectional study	56.9 ± 7.93	0.474	Self-report	NA	BMI ≥ 30	7
9	Mengxue 2018	0.69	0.54	0.88	Cross-sectional study	20–70	35.50%	Self-report	NA	NA	7
10	Megan E. Petrov	2020	USA	126	Cohort study	6 and 36 months	56.30%	Brief infant sleep questionnaire revised	NA	> 0.67 positive change in weight-for-age Z-score	7
11	SR Patel	2014	USA	6038	Cohort study	76.4/83.5	50.60%	Wrist actigraphy	> 1 h	BMI ≥ 30	9
12	Nan Wang	2020	China	14,685	Cross-sectional study	60.32 ± 9.66	47.60%	Self-report	< 1 h; > 1 h	BMI ≥ 28	8

*NA* not available.

### Quantitative assessment (meta-analysis)

#### Association between daytime napping and obesity risk

**Figure 2:**
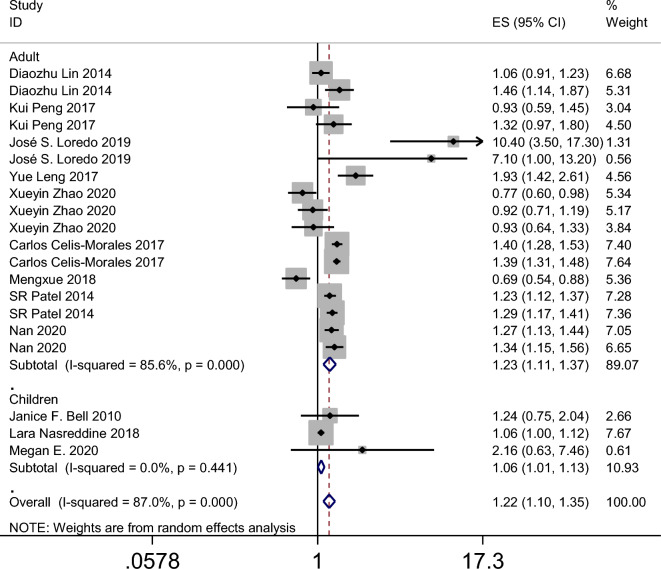
Forest plot evaluating the effect of daytime napping on the risk of obesity.

**Figure 3:**
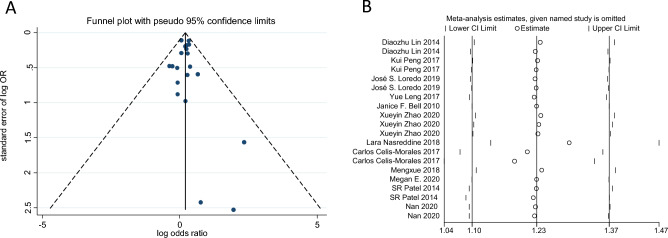
(**A**) Publication bias analysis based on a funnel plot. (**B**) Sensitivity analysis for the effect of daytime napping on obesity.

The meta-analysis revealed that individuals who took daytime naps had a higher risk of obesity compared to those who did not take naps, with an odds ratio (OR) of 1.22 (95% CI 1.10–1.35, *p* = 0.000, *I*^2^ = 87%) (Fig. 2). The funnel plot (Fig. 3A), Begg’s test (*p* = 0.626), and Egger’s test (*p* = 0.670) did not suggest any evidence of publication bias. However, significant heterogeneity was observed (*I*^2^ = 87%, *p* < 0.001). A sensitivity analysis indicated that the results were robust (Fig. 3B). In conclusion, the meta-analysis provides evidence that daytime napping is associated with an increased risk of obesity. However, the observed heterogeneity across studies suggests that further research is needed to understand the mechanisms underlying this relationship and identify potential confounding factors.

### Subgroup analyses

**Figure 4:**
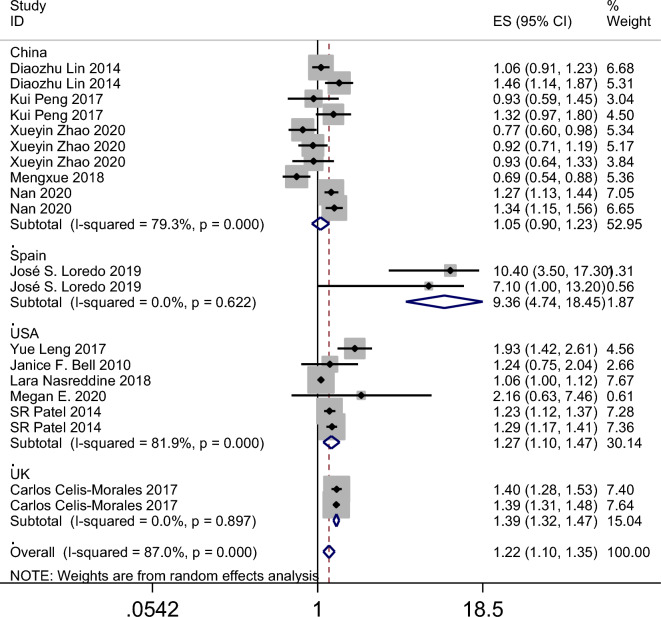
Forest plot evaluating the effect of daytime napping on the risk of obesity subgroup analysis stratified by country.

**Figure 5:**
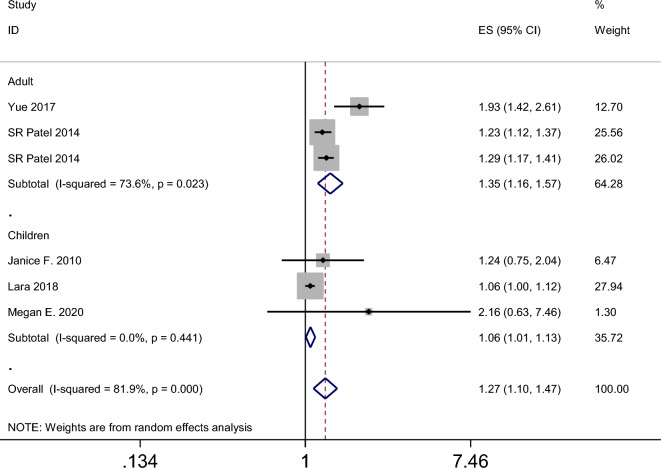
Forest plot evaluating the effect of daytime napping on the risk of obesity subgroup analysis stratified by age (Children or adults) in the American population.

**Figure 6:**
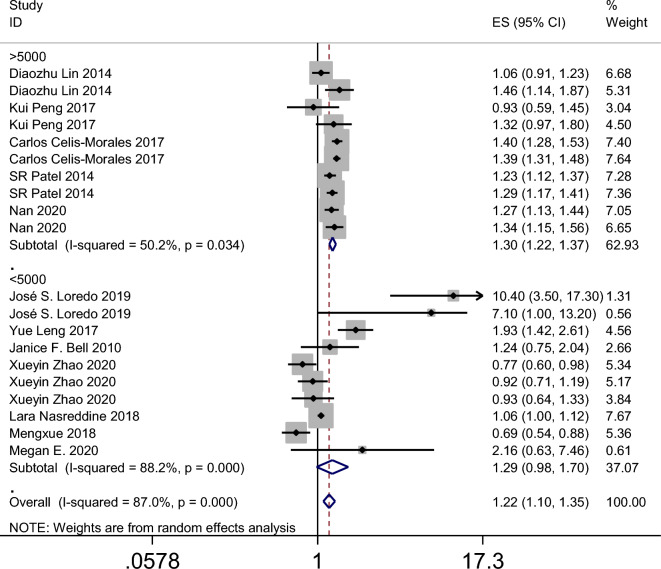
Forest plot evaluating the effect of daytime napping on the risk of obesity subgroup analysis stratified by sample size.

**Figure 7:**
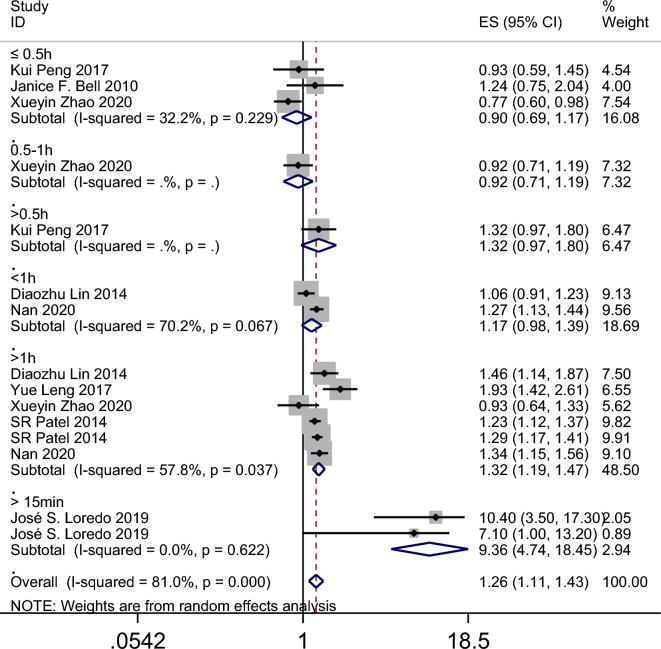
Forest plot evaluating the effect of daytime napping on the risk of obesity subgroup analysis stratified by nap duration.

**Figure 8:**
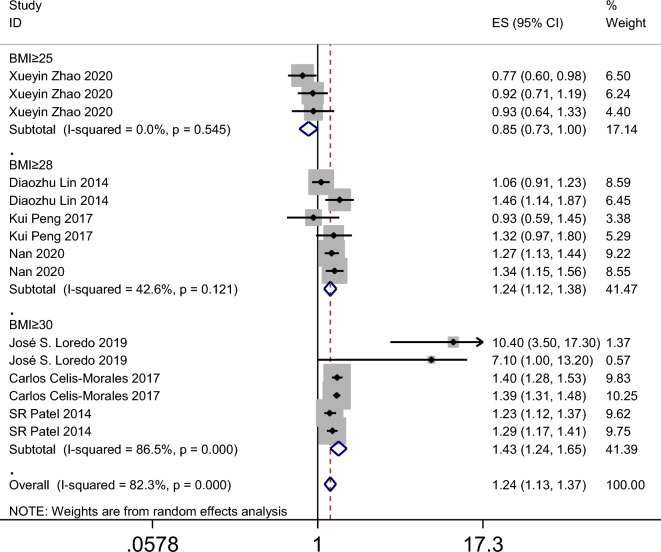
Forest plot evaluating the effect of daytime napping on the risk of obesity subgroup analysis stratified by definition of obesity.

The results of the meta-analysis revealed that the summary odds ratios (ORs) for the studies conducted in China, Spain, the USA, and the UK were 1.05 (95% CI 0.90–1.23), 9.36 (95% CI 4.74–18.45), 1.27 (95% CI 1.10–1.47), and 1.39 (95% CI 1.32–1.47), respectively (Fig. 4). The subgroup analysis based on age in the American population indicated that napping in both adults and child increased the incidence of obesity (Fig. 5). Additionally, the summary ORs for sample sizes > 5000 and < 5000 were 1.30 (95% CI 1.22–1.37) and 1.29 (95% CI 0.98–1.70), respectively (Fig. 6). The subgroup analysis based on nap duration showed an interesting finding: there was a significant increase in the occurrence of obesity when the nap duration was greater than one hour, whereas there was no clear relationship between nap duration less than one hour and obesity (Fig. 7). According to the sub-group analysis based on the definition of obesity, there was no significant relationship between napping and obesity when the diagnostic criteria for obesity were set at a BMI of greater than or equal to 25. However, when the diagnostic criteria were set at a BMI of greater than or equal to 28 or 30, napping was found to significantly increase the risk of obesity (Fig. 8).

## Discussion

### Main finding of this study

To the best of our knowledge, this is the first meta-analysis to investigate the potential link between daytime napping and obesity. Our findings suggest that daytime napping may play a significant role in the development of obesity. In this meta-analysis, which included twelve studies and up to 170,134 individuals, we found that daytime napping was associated with an increased risk of obesity. After conducting a subgroup analysis based on nap duration and the definition of obesity, an interesting finding emerged: when nap duration was greater than one hour, there was a significant increase in the occurrence of obesity, while there was no clear relationship between nap duration less than one hour and obesity. Moreover, when diagnostic criteria for obesity were set at a BMI of greater than or equal to 25, there was no significant relationship between napping and obesity. However, when the diagnostic criteria were set at a BMI of greater than or equal to 28 or 30, napping was found to significantly increase the risk of obesity. These findings suggest that the relationship between napping and obesity may be influenced by both the duration of the nap and the diagnostic criteria used to define obesity. Even after adjusting for bias and conducting sensitivity analysis, the association between daytime naps and obesity remained strong. Therefore, further research is needed to better understand the complex relationship between napping and obesity, and to determine the most effective strategies for reducing the risk of obesity in individuals who nap regularly.

### Mechanisms behind the association between daytime napping and obesity

The exact mechanisms underlying the association between daytime napping and obesity are still unclear. However, we have speculated on a few mechanisms that may help to explain this relationship.

First, daytime napping can activate the sympathetic nervous system (SNS), which has been associated with obesity. Therefore, daytime napping may influence obesity through the SNS^29^.

Second, daytime napping may extend bedtime, which could decrease thermogenesis and energy expenditure, potentially leading to obesity^30^.

Third, previous studies discovered that daytime napping and depression have a significant positive association and that more depressive symptoms are associated with diminishing oestradiol levels throughout the transition to menopause^31,32^. Moreover, a significant association between menopause and obesity has been found^33^. Therefore, menopause may have played a key part in the relationship between daytime napping and obesity.

Fourth, excessive daytime sleep may worsen insomnia at night, and fragmented sleep has been linked to higher BMI and increased risk of obesity^34^.

Fifth, circadian rhythm has a significant impact on body hormones, and recent research has found that excessive daytime napping is associated with elevated nighttime cortisol levels, which could increase insulin resistance and lead to abnormal blood lipids and fat distribution^35–37^.

Finally, daytime napping could be a sign of insufficient or poor sleep quality, which could be a contributing factor to the association between daytime napping and obesity^38^.

The study’s discussion of potential mechanisms underlying the association between daytime napping and obesity is speculative and based on existing literature. It is important to acknowledge that these mechanisms require further investigation and direct evidence to support their validity. These hypotheses help to illustrate the potential biological mechanisms underlying the association between daytime napping and obesity and provide clues for further exploration in this field.

### Strengths and limitations

This meta-analysis is based on the latest literature and is the largest comprehensive clinical study to date, with a sample size of 170,134. The advantage of our meta-analysis is the inclusion of a large population, making the outcomes more persuasive.

However, there are several limitations to consider. Firstly, the information on whether participants took naps and the duration of the naps was mostly obtained through questionnaire surveys, which may be influenced by sleep quality and emotion, potentially leading to obesity. To enhance the assessment of participants’ sleep patterns and quality, future studies could incorporate objective measures, such as actigraphy, to mitigate potential bias associated with self-reported data. Objective measures would provide more accurate and reliable information, leading to a more robust understanding of the relationship between daytime napping and obesity risk. Secondly, the majority of studies were conducted in China and the USA, with only one study each in Spain and the UK. Thus, the findings may not be generalizable to populations from other countries. Increasing the number of studies included in the analysis, particularly from a more diverse range of countries, would indeed enhance the generalizability of the findings. By including studies conducted in various cultural and geographical contexts, we can better understand how the relationship between daytime napping and obesity risk may vary across different populations. Thirdly, exploring the potential influence of lifestyle factors, such as diet and physical activity, on the association between daytime napping and obesity is an important avenue for future research. By considering these additional factors, we can better elucidate the complex interplay between napping behavior, lifestyle choices, and obesity risk. Fourth, it is essential to note that the current study cannot establish a causal relationship between daytime napping and obesity due to the limitations of the available data. Future studies should consider longitudinal designs or randomized controlled trials to provide stronger evidence for causality. Fifth, while the study assumes equivalence in the diagnostic criteria for obesity across studies, it is crucial to recognize that variations in how obesity is defined could influence the findings. Future studies should account for differences in obesity criteria and explore potential heterogeneity in the results. Finally, considering the potential influence of medication use and underlying medical conditions on both daytime napping and obesity is an important consideration. Future research should aim to gather relevant information on these factors to better understand their potential impact on the observed association.

## Conclusions

The results of this meta-analysis suggest that there is a positive association between daytime napping and the risk of obesity, independent of potential confounding factors. However, additional high-quality studies are needed to further elucidate the potential role of daytime napping in the development of obesity.

## Supplementary Information

Supplementary Information.

Supplementary Table 1.

## Data Availability

All data generated or analysed during the present study are included in this published article.
